# Experiences and perspectives of community health workers from implementing treatment for schistosomiasis using the community directed intervention strategy in an informal settlement in Kisumu City, western Kenya

**DOI:** 10.1186/s12889-016-3662-0

**Published:** 2016-09-15

**Authors:** Gladys O. Odhiambo, Rosemary M. Musuva, Maurice R. Odiere, Pauline N. Mwinzi

**Affiliations:** Neglected Tropical Diseases Branch, Centre for Global Health, Kenya Medical Research Institute, P. O. Box 1578-40100, Kisumu, Kenya

**Keywords:** Schistosomiasis, Mass drug administration, Community directed intervention, Community health worker, Informal settlement, Urban area

## Abstract

**Background:**

The Community Directed Intervention (CDI) strategy has been used to conduct various health interventions in Africa, including control of Neglected Tropical Diseases (NTDs). Although the CDI approach has shown good results in the control of onchocerciasis and lymphatic filariasis with respect to treatment coverage using community drug distributors, its utility in the control of schistosomiasis among urban poor is yet to be established. Using a longitudinal qualitative study, we explored the experiences, opportunities, challenges as well as recommendations of Community Health Workers (CHWs) after participation in annual mass drug administration (MDA) activities for schistosomiasis using the CDI approach in an urban setting.

**Methods:**

Unstructured open-ended group discussions were conducted with CHWs after completion of annual MDA activities. Narratives were obtained from CHWs using a digital audio recorder during the group discussions, transcribed verbatim and translated into English where applicable. Thematic decomposition of data was done using ATLAS.ti. software, and themes explored using the principle of interpretative phenomenological analysis (IPA).

**Results:**

From the perspective of the CHWs, opportunities for implementing CDI in urban settings, included the presence of CHWs, their supervisory structures and their knowledge of intervention areas, and opportunity to integrate MDA with other health interventions. Several challenges were mentioned with regards to implementing MDA using the CDI strategy among them lack of incentives, fear of side effects, misconceptions regarding treatment and mistrust, difficulties working in unsanitary environmental conditions, insecurity, and insufficient time. A key recommendation in promoting more effective MDA using the CDI approach was allocation of more time to the exercise.

**Conclusion:**

Findings from this study support the feasibility of using CDI for implementing MDA for schistosomiasis in informal settlements of urban areas. Extensive community sensitization and provision of incentives may help address the aforementioned challenges associated with implementing MDA using the CDI strategy. Opportunities highlighted in this study may be of value to other programmes that may be considering the adoption of the CDI strategy for rolling out interventions in the urban setting.

**Electronic supplementary material:**

The online version of this article (doi:10.1186/s12889-016-3662-0) contains supplementary material, which is available to authorized users.

## Background

Globally, more than 700 million people live in schistosomiasis areas and about 240 million people are infected [1]. It is estimated that about 800 million people are at risk of infection due to their exposure to contaminated water and 390−600 million people are infected with these parasites [2, 3]. Currently, the main control strategy for schistosomiasis is based on the periodic treatment of people living in at risk areas with anti-schistosomicidal drugs in order to reduce morbidity and transmission [4], with strategic scale up plans for mass drug administration (MDA) to control morbidity by 2020 [1]. For a long time, programs to control morbidity associated with infections of soil-transmitted helminths (STH) and schistosomiasis have depended largely on the delivery of anthelminthic drugs to primary schoolchildren because school-based deworming is advocated as a highly cost-effective public health intervention. However, a drawback of the school-based approach is that it does not reach children not attending school and other members of the community.

The success in achieving reduction in disease burden lies in ensuring that available health interventions reach those who are at risk. Many simple, affordable and effective disease control measures have had limited impact due to poor access especially by the poorer populations and inadequate community participation [5]. One proven strategy in addressing the problem of access to health interventions is the Community Directed Intervention (CDI), which is based on the principle of active structural community participation to prioritize and address their own health challenges [6]. The Community Directed Treatment with ivermectin (CDTi) used for mass distribution of ivermectin in rural African populations for the control of onchocerciasis and lymphatic filariasis [7–10] using community drug distributors (CDD) are good examples of programs that have successfully employed the CDI strategy. To improve access to treatment at affordable and sustainable costs and, where possible, to improve existing programmes, the CDI strategy has been presented as a viable alternative for control of schistosomiasis and STHs, and has been implemented in several settings including Kenya [11], Tanzania [12, 13], Mali [14] and Cameroon, Nigeria and Uganda [15], where CDDs and Community health workers (CHWs) also played a key role in treatment.

Although the CDI strategy has been piloted in Kenya [11] and community wide treatment for schistosomiasis has been established by several partners in Kenya [16] and other areas, these programs are mainly rural-based leaving out the urban areas where schistosomiasis thrives as well. Whereas majority of studies have been conducted in rural areas, it is noteworthy that there is limited data on the effectiveness of the CDI strategy in more challenging settings like urban informal settlements where the traditional community structure is non-existent. The clear socio-economic differences between rural and urban communities, and the need to investigate experiences of CHWs conducting MDA in urban areas has been indicated before [16]. Furthermore, there is a paucity of data on the experiences and challenges of CHWs in order to gain their perspectives on ways to improve MDA activities for schistosomiasis using the CDI approach in urban settings. In this longitudinal qualitative study, we explored the experiences, opportunities, challenges as well as recommendations of CHWs after participation in annual MDA activities for schistosomiasis using the CDI strategy in an informal settlement in Kisumu City, western Kenya. We highlight the implications of the challenges and suggest how they could be addressed, and propose opportunities that could be harnessed which may be of value to other programmes that may be considering the adoption of the CDI strategy for interventions.

## Methods

### Study area and population

The study was conducted in a sub-location (presented here as Area X to protect identity), an informal settlement in Kisumu City, western Kenya. The sub-location covers an area of 6.1 Km^2^ with a total population of 32,430 people, 16,189 of whom are male and 16,241 female [17]. The sub-location is headed by an Assistant Chief. There are five health units which are further sub-divided into nine sub-units, headed by one or two village elders depending on their size. The main economic activities in the area include fishing, car washing and small business enterprises. The main religion is christianity. Intestinal schistosomiasis is a public health problem in the area and its prevalence of 36 % is highest compared to all the other informal settlements in the City [18]. Poor sanitation prevails with high use of bush (open defecation) or ‘flying’ toilets (use of plastic bags for open defecation, which are then thrown into ditches, on the roadside, or simply as far away as possible). The lake is the main source of water for domestic use, but tap water, springs, boreholes, water vendors are also available.

### Community entry and stakeholder meetings

In order to create programme ownership, solicit support, galvanize interest and participation in the MDA, stakeholder meetings were held. Before the study commencement, the study team visited the sub-location and held a stakeholder meeting to find out which were the major health problems in the sub-location. Schistosomiasis was mentioned among HIV, typhoid and malaria. Since schistosomiasis was the focus of the present study, ways to control it were explored and mass treatment was suggested to be most cost effective intervention to be implemented, though health education, water and sanitation as well as mollusciding were also discussed.

Summaries of the study protocol were shared with different stakeholders including Ministry of Health officials, religious leaders, local politicians and administrators who were all sensitized on the benefits of the programme. Advocacy meetings were held at municipal headquarters targeting senior health officials. These officials helped in training drug distributors and later offered indirect supervision during the MDAs.

The programme embarked on different mobilization strategies to ensure uptake of the programme. First the local administration was used to sensitize the community through *barazas* (community gatherings), and through announcements at funeral ceremonies. Letters were written to religious leaders to inform their congregations about the programme and request for their participation. In addition, the programme conducted radio campaigns and road shows. The message was packaged in the three languages that the residents understood, *dholuo, Kiswahili* and English. Door to door campaigns were also conducted during the MDA. The community was also sensitized on the side effects associated with treatment and how to mitigate them.

### Training of community health workers

Training manuals were adopted from Ministry of health’s National school-based deworming program teacher training manual. These were edited by the programme officials to incorporate the programme’s needs and to make them suitable for CHWs. The training focused on schistosomiasis, praziqauntel (PZQ) distribution-related activities including community mobilisation, household enumeration, guidelines on the period and the duration of treatment, guidelines on the exclusion criteria, correct drug dosage using height, recognition and management of minor side effects, when to refer adverse reactions, basic record keeping, how to identify and report issues encountered during the distribution and how to report treatment coverage.

The programme designed a “cloth dose-pole” for PZQ dose determination based on height, which was easily portable for use during the MDA. CHWs were provided with treatment booklets, bags, T-shirts, respective dose poles and the drugs for distribution during the exercise. T-shirts served to identify the CHWs and as incentives to them, and bore deworming messages that aided in community sensitization. In addition, CHWs were given introductory letters for identification and as confirmation that they had been successfully trained to administer the drugs.

### Implementing the community directed intervention strategy

A stakeholders meeting was held with the community leaders to discuss various ways of schistosomiasis control. The community was involved in the planning, designing and implementing of the mass treatments. CHWs were proposed and agreed upon by consensus as drug distributors by the community. One village elder and 2 CHWs participated in treatment in each subunit. The CHWs selected for the exercise were identified from a pool of CHWs by the community members, on the basis of their commitment and hard work.

The duration for the study was three years, and refresher trainings on conducting MDA were provided to CHWs and village elders annually. During the first two years, the study team conducted MDA in the primary schools, but treatment in the schools was taken up by the government (through the National school based deworming programme) in the third year. The CHWs and village elders were given a daily allowance of Kshs 400 (USD 4) and Kshs 200 (USD 2), respectively, as an incentive to motivate them towards the MDA which was not part of their routine primary health care duties.

With the understanding that MDA is a public health intervention that can be implemented through a number of different approaches, CHWs were asked on their preferred mode of implementing the MDA. They proposed house-to-house administration of drugs, and this method was subsequently employed in the present study. Prior to implementation of MDA, CHWs conducted census for entire population of Area X that helped determine the population eligible for treatment and number of drugs required. Treatment was administered to eligible individuals according to WHO guidelines on preventive chemotherapy for schistosomiasis [1], based on their height determined using a cloth dose-pole. CHWs were encouraged to administer drugs after meal times (after breakfast, lunch or any other snack time). In most of the cases, CHWs directly observed swallowing of drugs, whereas in few other cases they left drugs behind to be swallowed once the household had a meal. For this study, children under 5 years, pregnant women and those who were sick were excluded from treatment. CHWs were supervised by Ministry of health’s Community Health Extensions workers (CHEWs) while the assistant chief supervised the village elders.

### Qualitative data collection, processing and analyses

Qualitative data collection was carried out in October of every year, from 2011 to 2013.

Unstructured open-ended group discussions with CHWs were conducted during the feedback sessions in each sub-unit after completion of the MDA every year. Four unstructured aide memoirs designed to promote open-ended responses were provided to stimulate the discussions [19]. The following aide memoirs were asked during the discussion: what were the CHW’s experiences and challenges faced during the MDA, what opportunities (if any) were available for CHWs to take advantage of, what were the perspectives of other community members regarding the MDA and what were the CHW recommendations on improving the MDA (Additional file 1). In addition to the general areas covered, the specific discussion direction was guided by how people responded to the aide memoirs, rather than by set questions.

Descriptive statistics were calculated for CHW demographic variables, socio-economic characteristics and amount of experience. The narratives during the group discussions were recorded using a digital audio recorder, transcribed verbatim in the native language of the study site of the participants (*Dholuo*), translated into English where applicable, and then verified for accuracy by another research assistant fluent in both *Dholuo* and English. Transcripts were then imported into

ATLAS t.i. qualitative data analysis software (Berlin, Germany) for analysis. Thematic decomposition of data and content analysis was done using a three phase coding system and following the principle of interpretative phenomenological analysis (IPA) as previously described [16]. The objective of IPA is to explore insiders’ opinions and beliefs related to their experience of a particular phenomenon [20]. Based on IPA, themes are considered to be direct representations of the phenomenon under study [21], an approach that provides an opportunity to explore social cognition and assumes that one’s interpretations of his or her experiences reflect the true nature of the phenomena [16], which in this case are the experiences and perspectives of the CHWs on the praziquantel MDA using the CDI strategy. Coding for identified themes was done using a three phase coding system (i) the primary researchers performed an initial scan of the transcripts that were imported into ATLAS t.i. qualitative analysis software, for the purpose of familiarization, highlighting words or phrases used by the participants, and establishing initial themes. Members of the research team identified the core themes through a process of collaborative analysis and linked the core themes to the aims of the study; (ii) the researchers focused on connecting themes and finding links in the data; and (iii) the primary researcher reread the data and assigned excerpts that illustrate the final themes. Narrative text was applied around the themes, with quotes used to illustrate the text and communicate its meaning to the reader. Decision trails were documented to guarantee that interpretations were supported by the data.

## Results

### Characteristics of CHWs

We described the CHWs by their demographic, socio-economic characteristics, and by number of trainings on health issues undertaken (Table 1). Of the 18 CHWs that participated in the study, 44.4 % (*N* = 8) were males and 55.6 % (*N* = 10) were females. The mean age of CHWs was 31.9 years; 72 % (*N* = 13) of the CHWs were married, 22 % (*N* = 4) were single, whereas 5.6 % (*N* = 1) were separated. All the CHWs attained some level of education; 78 %, (*N* = 14) attained secondary education and 22 % (*N* = 4) had attained postsecondary education. All the CHWs were christians and had over 3 health training (Table 1). At the time of the study, the government was not providing any form of payment to the CHWs.

**Table 1:** Socio-demographic characteristics of the 18 community health workers

Description	Frequency (*N* = 18)	Percentage (%)
Gender		
Male	8	44.4
Female	10	55.6
Age in years		
20-24	2	11.1
25-29	4	22.2
30-34	8	44.4
35-39	4	22.2
Educational level		
Primary education^a^	0	0
Secondary education^a^	14	77.8
College	4	22.2
Marital status		
Single	4	22.2
Married	13	72.2
Separated	1	5.6
Religion		
Christianity	18	100
Number of Health Trainings		
1-2 trainings	0	0
≥ 3 trainings	18	100
Occupation		
Volunteer work	7	38.9
Unskilled labour	3	16.7
Small business	8	44.4

^a^Attended primary or secondary education even if the level was not completed

### Experiences and perceptions by CHWs on MDA using the CDI strategy

Community members expressed different opinions about the MDA as noted and reported by CHWs during the group discussions. The experiences and perceptions by CHWs on MDA using the CDI strategy are examined using opportunities harnessed (what worked well/opportunities), challenges experienced by CHWs and their recommendations. A summary these experiences and perceptions is provided in Table 2.

**Table 2:** Summary of CHW experiences and perceptions on MDA in an urban setting

What worked well/opportunities • CHW familiarity with households led to warm reception • Good knowledge of intervention area by CHWs • High demand for drugs in the final year of treatment • Effective community mobilization • Opportunity to integrate MDA with other health interventions • Presence of CHWs and their supervisory structure, and points of referral for serious side effects
Challenges • Fear of side effects, size of tablet and misconceptions regarding treatment • Unrelated death and the associated negative publicity by the media • Religious beliefs and mistrust of interventions • Insufficient time and absence of community members during the MDA exercise • Difficulty in directly observing treatment • Unsanitary environmental conditions, insecurity and inaccessibility • Demand for incentives by community members to take drugs
Recommendations by CHWs • Increase in duration for conducting MDA and number of CHWs • Provision of incentives during the MDA exercise • Community sensitization through media • Incorporation of continuous Health education messaging in MDA exercises

- Opportunities explored by CHWs during MDA

CHWs experienced and took advantage of several opportunities to enhance their work during MDA. Some of the opportunities are highlighted below:

### CHW familiarity with households led to warm reception

Most of the CHWs had previously carried out health related activities in the households where they were administering treatment; they reported receiving a warm welcome in the households they visited. This was true even among the households that refused to take the drugs. However, one of the CHWs mentioned being chased out of a compound whose residents experienced side effects the previous year and claimed the CHW had come back to kill them. Members of the community seemed to be happy with the CHWs, but some only took issue with the drugs.

*“They did not have anything against us, it is our drug that they did not want. In fact they told us to return those family planning pills to whoever gave them to us. No amount of convincing would make them change their mind”*, said a male CHW.

Besides the fact that community members knew them, warm reception for CHWs was facilitated by sensitization about the treatment over the radio, road shows and also the fact that some of people knew the effects of bilharzia and were longing for an opportunity to get treatment. People who had suffered from bilharzia (hospital confirmed cases) before were easier to deal with.

*“There was good community reception since community members knew us, they heard about the treatment announced over the radio and some of them knew the effects of Bilharzia and were praying for such an opportunity”*, said a female CHW.

### Good knowledge of intervention area by CHWs

CHWs were knowledgeable about the area of intervention and this helped them move faster. CHWs also had the support of village elders who helped with identifying administrative boundaries between different sub-units.

### High demand for drugs in the final year of treatment

In the third year, all the CHWs reported that there was a higher demand for the drugs. This may have been related to people realizing that it was the final year of free treatment. CHWs reported people coming to demand for drugs at their houses during and even after the MDA. It is noteworthy that some community members encouraged CHWs to continue distributing the drugs after the final MDA. Community members were willing to participate in future MDAs.

### Effective community mobilization

CHWs appreciated that effective mobilization helped them go through some of the compounds faster. Most of the community members had already known about the MDA from the road shows that the programme carried out. Family members who got information about the MDA shared with other members of the household.

*“Awareness in some homes hence no time wasted due to many explanations”,* said a female CHW.

### Opportunity to integrate MDA with other health interventions

During the MDA, CHWs reported that some community members asked for other drugs like those for malaria and typhoid. Others also asked for bed nets, reporting that the ones they had were old and torn. Other people wanted medical checkups and the sick wanted treatment. CHWs referred the sick to hospital.

*“Other diseases apart from Bilharzia should be addressed. We should be given painkillers and drugs for malaria too to administer during the exercise”*, said a female CHW.

### Presence of CHWs and their supervisory structure, and points of referral for serious side effects

CHW infrastructure already exists in urban centres and they have a well developed supervisory structure by the Ministry of health. Community health extension workers offered supervision to the CHWs for free as part of their routine work, and the project did not have to remunerate them.

The local health centre (in Area X) helped in the management of adverse side effects of PZQ, and this helped in reassuring the community that all was well. The health facility in addition to managing side effects helped in sensitization by letting the community members who came to the hospital understand the benefits of treatment.

b. Challenges in Implementing MDA using the CDI strategy

The challenges noted by CHWs during the MDA exercise are highlighted below:

### Fear of side effects, size of tablet and misconceptions regarding treatment

Some people were unwilling to take drugs because they had already heard from their neighbours who had received drugs complain about side effects. Since treatment was conducted over a period of time (initially 7 days then 12 days), those who had seen their neighbours experience side effects did not want to take the drugs. Furthermore, those who experienced side effects in the previous year did not want the drugs in subsequent MDAs. The main side effects reported included: fatigue, stomachache, vomiting, dizziness, itchiness and diarrhoea. On the other hand, some people who were willing to take the drugs complained that PZQ tablets were too big to swallow. A male CHW reported that one household head told him,

*“My people and I will not take those drugs of yours. Never! In fact leave my compound! Those drugs almost killed me last year; I had terrible diarrhoea and headache. I could not even fend for my children. I will never take those drugs again”.*

CHWs explained that it was difficult to convince those who had previously experienced side effects to take the drugs again. CHWs also found it hard to convince those who had no knowledge of the disease to take the drugs.

Some people declined to take drugs because they misconceived schistosomiasis to be a disease caused by promiscuity. They therefore felt that since they were faithful to their partners and their children were not sexually active, treatment was not necessary.

*“They believed that bilharzia is a disease caused by promiscuity and since they are faithful and their children are young they do not need the treatment at all”*, said a male CHW.

In addition, other people believed that treatment was not necessary since they did not feel sick at the time of MDA. A female CHW reported that a member of the community told her,

*“I do not need those drugs of yours, I am not feeling sick”*.

Those left out of treatment because they had other underlying conditions such as epilepsy or were pregnant felt they were being discriminated against, and in some cases would refuse to let other family members take drugs.

### Unrelated death and the associated negative publicity by the media

Negative publicity by the media was a major challenge in the third round of MDA. Just before we conducted our MDA, treatment through the National school based deworming programme had been implemented, and unfortunately a child died during the deworming exercise in Kisumu. Though the death was not related to the deworming exercise, the timing of the death coupled with sensational reporting in one of the local dailies (http://www.nation.co.ke/counties/kisumu/Pupil-dies-in-deworming-drive/-/1954182/1877556/-/view/printVersion/-/qb425a/-/index.html) led the community to erroneously associate it with the deworming drugs. The death and negative publicity greatly affected treatment compliance in the third year of the study, and lots of effort had to be put in place to clear the misunderstanding.

*“Bad publicity by the media about the school child who died during deworming in schools made people fear the drug”*, stated a male CHW.

### Religious beliefs and mistrust of interventions

Due to their religious beliefs, some households did not take drugs. When they fall sick they pray and hope to get better, and under no circumstances are they allowed to take medicines. Other households did not want to take the drugs clamming that it was a government’s programme to roll out a family planning initiative that would make them and their children infertile. Other community members also thought that since the drugs were being offered for free, then there was a likelihood that the drugs were either not genuine, had expired or that they (community members) were being used as ‘guinea pigs’ to test the drugs. Interestingly, some had a strong belief that nothing is for free in this world.

*“Some community members felt that they were just being used as guinea pigs and considered the MDA an experiment”*, reported a male CHW.

Some people also resisted treatment because they had not provided samples for testing. CHWs spent an appreciable amount of time explaining to them the principle behind preventive chemotherapy for at risk populations, and convincing them to take the drugs.

### Insufficient time and absence of community members during the MDA exercise

CHWs complained unanimously that the time allocated for the MDA was inadequate.

*“The time the programme has allocated for treatment is too brief. I think it should take a minimum of two weeks because the subunits are large and have many people”*, said a female CHW.

In the first year, MDA was implemented within one week and during working hours. From the annual feedback, it was noted that those who were out at work missed out on treatment. Therefore, it was recommended that subsequent MDA be conducted inclusive of weekends, and this resulted in better treatment coverage. However, some people still missed treatment since they left too early in the morning for work and came back too late in the night. A female CHW reported that,

*“There were fishermen and other people who left very early and come back too late in the night because of work. Even during the weekend they were still out working. Me and my village elder came back to the homestead many times but we did not find them and neighbours told us some of them came back at midnight and would leave by 5 am. So there was nothing i could do”*.

*“Houses with members out on other errands and the like could not be found completely. Some of the people that had been registered could not be found during the treatment exercise. This is after making several trips during the day, at noon and even at night”*, reported a male CHW.

### Difficulty in directly observing treatment

During training for the MDA, it was emphasised that Direct Observed Treatment (DOTs) should be carried out. However, all the CHWs together with their village elders pointed out that this was not possible. CHWs had been advised to treat after meals, and this proved difficult to achieve among these urban poor where a meal was not guaranteed every day. Moreover, community members preferred to take drugs at certain times, especially at night, forcing CHWs to go back and forth in order to observe the swallowing of the tablets. Despite this effort, CHWs still had to leave the drugs behind in some cases, with the assurance that the drugs would be swallowed after dinner. CHWs also lamented that treatment after meals was difficult since most people could only have one meal in a day, mostly dinner, which was served very late in the night to avoid unwanted guests coming to partake as well.

*“Community members preferred to take drugs at certain times especially at night after late dinner to keep away hungry neighbours”*, reported a male CHW.

### Unsanitary environmental conditions, insecurity and inaccessibility

Some areas were too filthy and bushy, whereas some neighbourhoods were insecure. Since open defecation and fly toilet is widely practised in this area, CHWs were scared of navigating the bushes to get to some households as they did not have protective footwear like gumboots. The unsanitary conditions in the study area were aptly captured by these two CHWs:

*“You know in (Area X) people just defecate anywhere especially where they see bushes. So if you see a bush you cannot step on it with your sandals to reach that household. That is why we need gumboots. It is filthy and pathetic”*, said a male CHW.

***“****People do not have toilets. Many landlords do not construct toilets so fly toilet is the order of the day. It is cheap also to get a house in a place where they is no toilet so many people go for that because they are poor”*, reported a female CHW.

Just like other informal settlements, the study area is plagued by insecurity. A female CHW reported,

*“I felt insecure in some of the places I tried to visit late evening to catch up with members who could not be found during the day”*.

CHWs reported that some of the households were also inaccessible due to floods that had swept away wooden bridges, making it difficult and dangerous to access some households.

### Demand for incentives by community members to take drugs

Since members of the community were asked to take the drugs after a meal, some demanded money from the CHWs to facilitate purchase of food. Some mentioned that they had not eaten anything for the last couple of days and requested the CHW to buy them flour for porridge. On the other hand, some people thought that the CHWs were paid a lot of money (depending on the number of people they treated), and demanded their own share of the CHW’s money on the grounds that they were helping the CHW earn a bigger pay by accepting to take “their” drugs.

c. Recommendations by CHWs on how to improve MDA using the CDI strategy

CHWs provided recommendations that they felt could help strengthen future MDA exercises using the CDI strategy in urban areas. Their recommendations included:

### Increase in duration for conducting MDA and number of CHWs

According to the CHWs, the initial one week allocated for treatment was inadequate and recommended that in future more time should be allocated. They felt that two weeks could be sufficient. They further recommended that treatment should be organized in late evenings or over weekend to reach those who go to work. Treatment over the weekend was organised and implemented in the second and third year of MDA in the present study. However, treatment late in the night could not be implemented due to concerns over insecurity. They also suggested that more CHWs should be involved in the exercise in the future, proposing 4 per sub-unit instead of 2 as was designed in the present study.

### Provision of incentives during the MDA exercise

The CHWs appreciated the modest daily allowance provided by the study, but requested that an additional lunch allowance be included since they had to walk the whole day during the MDA and did not get time to go back to their own houses for lunch. They appreciated the T-shirts that were provided by the study, but requested that in the future they should be provided with more than one T-shirt to facilitate change of clothes. Some CHWs felt that a snack should be availed in the community, as is the practice for some studies conducted in schools. They also wished that future MDAs should provide gumboots and umbrellas to CHWs to facilitate their work during floods and rainy season.

*“Our people are very poor; the project should provide porridge in the community like they do in schools. Imagine someone telling you that they have not eaten for three days and you are here insisting that the drug should not be taken on an empty stomach! It is very sad”*, said a female CHW.

### Community sensitization through media

CHWs recommended that massive media campaigns through local radio stations should be employed during all treatment seasons.

### Incorporation of continuous health education messaging in MDA exercises

CHWs mentioned that community members still needed extensive health education throughout the duration of MDA so that they would be properly informed about schistosomiasis. They suggested that health education messages should entail not only the importance of treatment but also encourage people to construct and use latrines for waste disposal.

*“Health education should be held in the community so that as they get treated they also learn about importance of building latrines”*, said a male CHW.

## Discussion

This is one of the few studies that have documented the experiences of CHWs participating in MDA using the CDI strategy in an urban setting. Despite several challenges pointed out, findings indicate the utility of the CHW infrastructure in rolling out MDA in an urban informal settlement where the traditional community structure is non-existent, and highlights various opportunities that can be harnessed. It should be acknowledged that using CHWs to obtain the views of the community members is an indirect approach and may not fully represent the perspectives of the individuals receiving treatment. However, CHW involvement presents opportunities for community-based participatory program evaluation because of their unique position as a bridge between health systems, program managers, and the communities they serve [16]. As a result of CHWs participation, program managers are able to integrate useful feedback from communities to solve challenges that arise during implementation of and monitoring of intervention programs.

Community participation in primary health care has been hailed in the Alma Ata declaration as the key to universal goal of health for all [22]. Its application in rural areas has been extensively studied but little attention has been paid to its application in underserved poor urban areas. One of the proposed avenues to ensuring coverage of health interventions for all is through the CDI strategy, which builds upon the core principles of primary health care. The CDI strategy has been evaluated and found to be highly successful in reaching persons at risk of disease within the community [23], and more specifically, to control schistosomiasis and STHs in various endemic areas in Africa [12–15]. In Kenya, the CDI strategy was successfully implemented in the rural areas for the control of schistosomiasis [11], but the strategy had not been tested in an urban setting. The present study has shown that the CDI strategy can be implemented in urban areas for schistosomiasis control. The entry strategy where CHWs were recommended by community representatives during the stakeholder meeting and the members got to prioritize and take charge of their health problems were key in ensuring successful implementation of the MDA. Not only did community members decide on the most appropriate mobilization techniques, but also the on the timing of the MDA, the method of distributing drugs and the composition of teams and allocation of chores between the CHWs and village elders. The present study only offered training for the CHW to ensure that the drugs were distributed in accordance with WHO guidelines. More often than not, ccommunity involvement, ideally steered by communities to address their own priorities, is often initiated and driven by external agencies. These agencies usually focus on outputs rather than the quality of processes, and they are not necessarily inclined to let go of authority and influence [24]. Ownership of programs by the community and empowerment leads to both increased acceptability and sustainability.

Despite the successful implementation of MDA in our study, several challenges emerged. As observed in this study and elsewhere [14], fear of side effects, experienced by self, observed or from hearsay can lead to refusal of treatment. In north-western Uganda, Parker and others [25] reported that fears of side-effects of PZQ, which were not addressed by the treatment programme, negatively affected the national schistosomiasis and STH control programme. Fear of side effects is further confounded by the relatively large size of PZQ tablets. The reported misconceptions regarding treatment are not unique to our study, and are consistent with findings from a study in rural Kenya [16] and in Uganda [25]. The co-occurrence of unfavourable events such as deaths during MDA is another significant factor that may impact a control programme. The biggest test that can define the outcome of MDA is how information relating to such unrelated negative events is managed. Negative publicity by the media for the death of a child during the national deworming exercise negatively affected the MDA exercise in the present study. Despite the fact that deworming drugs are safe and not known to cause deaths [26], such rare and unrelated events when they occur are often accompanied by huge negative media publicity, as was recently evidenced in India (http://www.thehindu.com/news/national/other-states/death-of-girl-due-to-pneumonia-not-deworming-pill-says-centre/article8246090.ece). Sensitization of different stakeholders on importance of accurate reporting and management of information would certainly mitigate such negative publicity. On the other hand, misconceptions and mistrust for research and in some cases for government interventions have been reported in sub-Saharan Africa since colonial times [27]. For some while, blood collection, regular treatments (such as vitamin supplementation or malaria prophylaxis) and interventions targeting specific age or gender groups (such as vaccination and family planning), have been suspected of reducing the fertility of young girls [28, 29]. Findings from a recent study in western Kenya also reported some level of mistrust towards the government by community members [16]. Such suspicions and misconceptions by community members if not properly addressed, have the potential to impede recruitment into research, affect adherence to or even threaten continuation of health interventions. Intensified community sensitization and providing additional information regarding the intervention often helps to create more understanding and acceptability.

Other challenges noted in our study were insecurity, demand for incentives and lack of awareness on schistosomiasis. Insecurity hampered access to certain neighborhoods or working late in the evenings by CHWs, consequently circumventing CHWs from reaching their full potential. Implementation of MDA, just like any other health intervention, cannot be effectively carried out where insecurity thrives. As a stakeholder in the health of their citizens, governments may boost health interventions through ensuring provision of security to all, regardless of their class in society. Security is therefore paramount if CHW-supported programs are to continue expanding access to basic health services. In contrast to previous findings that community implementers were more motivated by intangible incentives than by external financial incentives [15], demand for financial incentives (both by CHWs and community members) appeared to be pronounced in our study. In Mali, Dabo and others [14] reported that CDDs dropped out due to lack of incentives. However, it is noteworthy that although incentives may serve to motivate CHWs and improve their performance, this is not always the case as evidenced from previous findings that showed no significant relationship between the provision of incentives to CDD and treatment coverage [7]. On the other hand, it has been argued that financial or other incentives tend to lead to over-reported coverage. Considering that informal settlements are inhabited mainly by the urban poor, high demand for incentives observed in our study was not unexpected. Polarized discussion continues globally about how to reward CHW performance and whether CHWs should be paid. Whereas some CHWs prefer extrinsic benefits, it has been observed that some CHWs are motivated by intrinsic factors such as the social status associated with the job (being referred to as “doctor”), recognition by the local community members, trainings and certificates [16]. The downside of the title “doctor” is that it may lead to abuse of trust from communities if CHWs provide health care services that they are not authorized or qualified to offer. There is clearly a need to emphasize community ownership for interventions and to de-emphasize incentives for CHWs and community members in such settings. At the same time, retention of CHWs without financial incentives may present a challenge for NTD programs, especially when other programs such as those for malaria and HIV/AIDS are providing incentives. In the present study, CHWs observed higher decline for treatment among members of the community who were not aware of schistosomiasis, and this is consistent with studies from Uganda [30], Tanzania [31] and Cote d’Ivoire [32]. We have previously reported low awareness of schistosomiasis in this study area [33]. Community sensitization through health education on the disease, importance and benefits of deworming will increase acceptance of treatment.

Several opportunities were highlighted by CHWs that could be harnessed to improve MDA using the CDI strategy. The availability of CHWs who had already been trained by partners such as Non-governmental organization (NGOs) and their associated supervisory structures including CHEWs from the government was resourceful. We found out that these CHWs were already collecting data on day to day health status of community members, providing monthly reports on immunization, malaria, bed net distribution, TB and HIV interventions. However, deworming for schistosomiasis was not part of their routine work. The unique role played by partners such as NGOs in complimenting government efforts may also strengthen the CDI strategy. A similar observation was made by Ajayi and others [34] who also found that NGOs conduct health education on various health issues, and train volunteers such as peer educators, change agents and implementers of interventions and therefore recommended the use of NGOs in implementing CDI programmes. Several other authors have demonstrated that community-based healthcare providers who comprise of traditional birth attendants, patent medicine sellers, paramedics, lay mother volunteers, role model caregivers for home management of malaria (RMC) and other community-oriented resources persons (CORPs) have the ability to provide effective community/home-based treatment of malaria, which subsequently reduced morbidity and mortality from the disease [35, 36]. As noted by Ajayi and others [34] and echoed by findings in the present study, human resources for health are an important element in the CDI process. In our study, the human resource component was augmented by the presence of points of referral for serious side effects. More often than not, while the urban rich areas have access to modern healthcare services as they are served by secondary and tertiary institutions, the urban poor areas have limited and unequal access [34]. Therefore opportunities in the community need to be explored to enhance access to health facilities and ensure health equity for all members of the community. While community-based interventions have been found to be more equitable than facility-based services, programs must still ensure that underserved or disadvantaged areas receive access [37].

As noted in the present study, familiarity of CHWs with households, their knowledge of intervention area and effective community mobilization represent other opportunities to be tapped for successful implementation of MDA using the CDI strategy. Context-relevant community awareness campaigns that built local ownership of the programme are known to result in high levels of acceptability of programmes and participation by the community [38], as opposed to purely “top-down” approaches to programmes. The other opportunity noted relates to integration of MDA with other primary-healthcare services or health interventions. The common overlap of many of the diseases opens opportunities for integrated provision of interventions through leveraging of their respective physical infrastructures and human resources and maximizing coverage, while reducing the overall cost of the programme. The demand for other interventions alongside MDA was noted in the present study. Nevertheless, incorporation of such additional horizontal interventions and devolution of tasks to CHWs requires careful planning so as not to compromise the initial objectives of the primary intervention.

## Conclusions

The present study has shown that the CDI strategy can be used for successful implementation of MDA for schistosomiasis in an urban setting. Lack of motivation and incentives for CHWs could be a major obstacle to their sustainability and consequently the effectiveness of the CDI strategy. CHWs would appreciate some form of incentive or compensation for their work. Despite the challenges highlighted, the CDI strategy also presents, at all stages of its implementation in the urban setting, opportunities for the integration of other health interventions.

## Additional file

Additional file 1: Unstructured open-ended Group discussion Guide for discussion with Community Health Workers. Community Directed Intervention for Schistosomiasis and Soil-transmitted helminth (STH) infections in an urban setting, western Kenya. Unstructured open-ended Group discussion Guide for discussion with Community Health Workers. The file contains unstructured open-ended key questions that were used to guide discussion with community health workers that participated in MDA exercise during feedback sessions. (DOCX 13 kb)

## Abbreviations

CDD: Community drug distributors
CDI: Community directed intervention
CHW: Community health worker
FGD: Focus group discussion
IPA: Interpretative phenomenological analysis
KShs: Kenya shillings
MDA: Mass drug administration
PZQ: Praziquantel
STH: Soil transmitted helminths
USD: United States dollar
