# Supplementary material for: Experiences and perspectives of community health workers from implementing treatment for schistosomiasis using the community directed intervention strategy in an informal settlement in Kisumu City, western Kenya
Source: BMC Public Health. 2016 Sep 15;16:986. doi: 10.1186/s12889-016-3662-0 (PMC5025566; doi:10.1186/s12889-016-3662-0)
Supplement: Additional file 1: — Unstructured open-ended Group discussion Guide for discussion with Community Health Workers. Community Directed Intervention for Schistosomiasis and Soil-transmitted helminth (STH) infections in an urban setting, western Kenya. Unstructured open-ended Group discussion Guide for discussion with Community Health Workers. The file contains unstructured open-ended key questions that were used to guide discussion with community health workers that participated in MDA exercise during feedback sessions. (DOCX 13 kb) [file 12889_2016_3662_MOESM1_ESM.docx]

**Title: Community Directed Intervention for Schistosomiasis and Soil-transmitted helminth (STH) infections in an urban setting, western Kenya**

**Unstructured open-ended Group discussion Guide for discussion with** **Community Health Workers**

**Who to interview:** Community health workers that participated in MDA exercise

**When:** Administer instrument after MDA exercise during feedback sessions

**Introduction:**

Good day. I am ….…..... I’m from …......and wish to learn about your thoughts on the just completed MDA exercise. The objective of this session is to collect information to help improve this activity next year. We will talk to you for about 1 hour. We will record our discussion with you using a voice recorder to allow us to accurately capture the information you provide. Your name and what you say to us during this interview will be kept confidential. Participation in this interview is voluntary. Whoever is not willing to participate may leave the session now or at any time during the discussion.

Do you have any questions about this discussion? If you have any questions about your rights in the study or any other issue, you may contact...........................

**Key questions to stimulate discussion:**

1. What are the experiences and challenges you faced during the MDA exercise?
2. What opportunities did you take advantage of?
3. What are the perspectives of other community members regarding the MDA?
4. What recommendations do you have to help improve the deworming programme?
